# The Donkey Genome: From Evolutionary Insights to Sustainable Breeding Strategies

**DOI:** 10.3390/ani16010093

**Published:** 2025-12-29

**Authors:** Qifei Zhu, Muhammad Zahoor Khan, Yadi Jing, Mingyang Geng, Xuemin Zhang, Yunfan Zheng, Xianggang Cao, Yongdong Peng, Changfa Wang

**Affiliations:** 1 Liaocheng Research Institute of Donkey High-Efficiency Breeding and Ecological Feeding, College of Agriculture and Biology, Liaocheng University, Liaocheng 252000, Chinazahoorkhan@lcu.edu.cn (M.Z.K.);; 2 Yili Kazak Autonomous Prefecture Livestock General Station, Yili 835000, China

**Keywords:** donkey genome, population genomics, production traits, breeding, conservation

## Abstract

Donkeys (*Equus asinus*) are economically and ecologically important livestock species whose genetic potential remains underexplored. This review synthesizes recent advances in donkey genomics, tracing their evolutionary history while evaluating current applications in selective breeding, conservation genetics, and agricultural management. By integrating evidence from population genomics, functional genomics, and comparative evolutionary studies, we summarize major genomic discoveries and identify persistent knowledge gaps, with a focus on translating genomic information into practical breeding outcomes. High-quality reference genomes, population resequencing, and ancient DNA analyses have clarified the African origin, global dispersal history, and environmental adaptation of donkeys. Genome-wide approaches, including GWAS, QTL mapping, and multi-omics analyses, have further identified genes and regulatory pathways associated with thermotolerance, metabolism, reproduction, and milk production. Nevertheless, progress is still limited by small sample sizes, variable sequencing depth, and inconsistencies in phenotyping and bioinformatic pipelines, which constrain cross-population comparisons and practical applications. Addressing these challenges through standardized phenotyping, improved data integration, and collaborative research frameworks will lay the groundwork for effective conservation strategies and sustainable genomic breeding of global donkey populations.

## Simple Summary

Donkeys are an important livestock species that play a significant role in agriculture and rural livelihoods. However, their genetic potential has remained largely unexplored. Recent advances in genomics have provided new insights into the evolutionary history of donkeys. These studies have also identified genetic factors underlying key traits related to survival and productivity, including heat tolerance, metabolism, and reproduction. Such findings are essential for improving breeding programs. They can enhance donkeys’ adaptability to diverse environments and optimize their contributions to milk, meat, and hide production. Despite these advances, the application of genomics in donkey breeding is still constrained. Small sample sizes and inconsistent data quality remain major challenges. This research proposes a roadmap to overcome these limitations. It emphasizes improved data collection, optimized breeding strategies, and sustainable conservation efforts. By addressing these issues, the study aims to support the long-term preservation of donkey populations and maximize their economic value for future generations.

## 1. Introduction

The donkey (*Equus asinus*), one of the earliest domesticated equids, has supported human societies for thousands of years [1]. Originating from African wild ancestors, donkeys dispersed across Eurasia and became integral to transport systems, agricultural production, and long-distance trade networks [2,3]. Today, donkeys remain essential for draft power and livelihood support in many developing regions. At the same time, their economic importance in milk, meat, and hide production is steadily increasing [4,5,6,7,8,9,10,11]. Biologically, donkeys exhibit remarkable resilience to arid environments, efficient metabolic regulation, and flexible reproductive performance. These characteristics make them a valuable model for studying adaptation and domestication within the genus *Equus* [12,13].

Global donkey populations are currently experiencing profound demographic and socio-economic changes. Mechanization and reduced dependence on animal traction have caused sharp population declines in many low- and middle-income countries [14]. In parallel, the rapid expansion of the ejiao industry has driven a surge in demand for donkey hides, further accelerating population losses and unregulated slaughter [15]. Together, these pressures threaten the genetic integrity of indigenous donkey breeds and undermine the livelihoods of pastoral and smallholder communities [16,17]. In contrast, intensive and semi-intensive breeding systems are emerging in China and parts of Europe, particularly for donkey milk production. Donkey milk is valued for its compositional similarity to human milk and its hypoallergenic properties [18,19,20,21,22,23]. These contrasting global dynamics highlight the urgent need to conserve genetic diversity while simultaneously improving productivity through genomics-informed and sustainable management strategies [24].

Over the past decade, technological innovation has fundamentally reshaped donkey genomics research [25]. Early draft genome assemblies provided initial insights into genome organization but were often fragmented and limited in functional annotation [26]. More recently, advances in long-read sequencing platforms, including Oxford Nanopore Technologies (ONT) and PacBio HiFi sequencing, together with Hi-C scaffolding, have enabled the generation of chromosome-level assemblies [27]. In parallel, population resequencing and ancient DNA analyses have clarified domestication processes, geographic dispersal routes, patterns of genetic diversity, and signatures of environmental adaptation [12,26,27,28,29,30,31]. As a result, high-quality donkey reference genomes have become available, creating new opportunities to investigate the genetic architecture of complex traits and to support evidence-based breeding and conservation programs [32,33,34,35,36].

Despite significant advances, several important challenges remain in donkey genomics. Compared with major livestock species, foundational insights into the genetic architecture of adaptive and production-related traits are still emerging. The genomic basis of reproductive traits, critical for population sustainability and breeding efficiency, has only recently received systematic attention. Integration of genomic data with standardized phenotypic and environmental information remains limited, and the practical translation of genomic findings into routine breeding and conservation strategies is still in its early stages. Nevertheless, recent studies have made substantial progress, providing key discoveries in evolutionary history, trait biology, and candidate gene identification. Building on these advances, this review summarizes major developments in donkey genomics, highlights important breakthroughs, identifies remaining knowledge gaps, and outlines future directions for sustainable breeding, effective conservation, and responsible industry development.

## 2. Materials and Methods

This review followed a structured literature-based approach to summarize advances in donkey genomics, genetic diversity, and trait-related biology. Studies published between 2000 and 2025 were primarily retrieved from the Web of Science Core Collection, chosen to ensure coverage of peer-reviewed SCI literature, with supplementary searches conducted in PubMed, Scopus, Elsevier, and Google Scholar. Search terms were applied using combinations of keywords related to donkey (*Equus asinus*), genomics, genetic diversity, domestication, phenotypic traits, adaptive evolution, and multi-omics, linked by Boolean operators (AND/OR).

Articles were screened through title, abstract, and full-text evaluation. Inclusion was limited to peer-reviewed studies reporting primary genomic or phenotypic data or providing comprehensive analytical syntheses relevant to breeding, adaptation, or conservation. Conference papers and book chapters were excluded. Non-SCI sources were generally excluded to ensure data reliability, although recent high-impact preprints were examined qualitatively to capture emerging trends when consistent with peer-reviewed evidence. In total, more than 420 articles were initially screened, and 161 studies were ultimately included in this review. The final set of studies formed the basis for the synthesis and interpretation presented in this review.

## 3. The Construction and Optimization of the Donkey Reference Genome

### 3.1. Evolution of Genome Assembly Technologies in Donkeys

Early donkey genome assemblies, initiated around 2015 and built primarily with short-read sequencing platforms, provided essential but fragmented references that limited the resolution of structural variants and gene organization [26]. Despite these constraints, these early efforts laid the groundwork for comparative analyses with other equids and offered preliminary insights into donkey evolutionary history. A major improvement was achieved through proximity ligation approaches such as the Chicago/HiRise system, which substantially enhanced scaffold continuity and enabled the detection of chromosomal rearrangements, providing early evidence of karyotypic divergence within *Equus* [28]. Subsequent assemblies, including the Texas donkey genome, further improved assembly completeness and annotation accuracy, establishing a stable foundation for studies of population differentiation and domestication [26].

**Figure 1:**
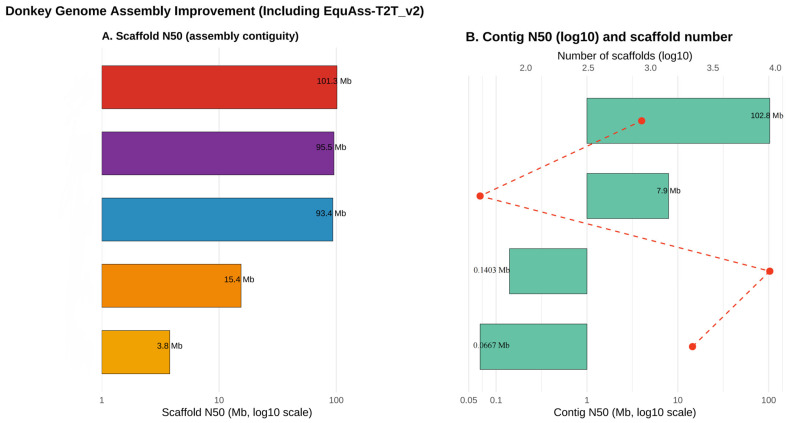
Comparison of donkey genome assembly quality improvement. The data, derived from references [26,27,28,44,45]. (**A**) Scaffold N50 values (Mb, log10 scale), where colored bars represent different genome assembly versions (reference numbers in brackets). (**B**) Contig N50 values (Mb, log10 scale; green bars) and scaffold numbers (log10 scale; red dots). Red dotted lines connect scaffold numbers to their corresponding assemblies, illustrating changes in assembly contiguity and fragmentation.

More recently, the integration of advanced sequencing and scaffolding technologies has enabled a transition from reference-quality drafts to true chromosome-level assemblies [37]. The incorporation of long-read sequencing platforms (PacBio SMRT and Oxford Nanopore), together with Hi-C chromatin conformation capture and optical mapping, has allowed accurate haplotype phasing and the reconstruction of structurally complex genomic regions [38,39]. For the first time, repetitive regions such as centromeric and subtelomeric sequences could be reliably assembled, revealing lineage-specific rearrangements and mechanisms underlying hybrid incompatibility between donkeys and horses [40,41,42,43]. These cumulative advances, summarized in Figure 1, have fundamentally transformed the resolution achievable in donkey genomics and now serve as the cornerstone for high-resolution comparative analyses and for exploring the genomic basis of reproductive isolation, karyotype evolution, and species adaptation.

### 3.2. Multi-Omics Integration for Functional Genome Annotation

Building upon these robust assemblies, the integration of multi-omics data has shifted the focus from genome structure to function. Combined RNA-seq, Iso-Seq, and ATAC-seq datasets have expanded gene models, resolved alternative splicing, and mapped cis-regulatory landscapes across tissues [46,47,48]. These efforts have revealed the distribution and regulation of protein-coding and non-coding RNAs, while long-read structural variant analyses have pinpointed genomic regions associated with domestication, adaptation, and production traits [30,49]. Together, these developments represent a conceptual transition in donkey genomics—from constructing the genome to interpreting its biological meaning—providing the framework for connecting genotype to phenotype and for guiding future breeding and conservation programs.

## 4. Population Genomics and Domestication History of Donkeys

**Figure 2:**
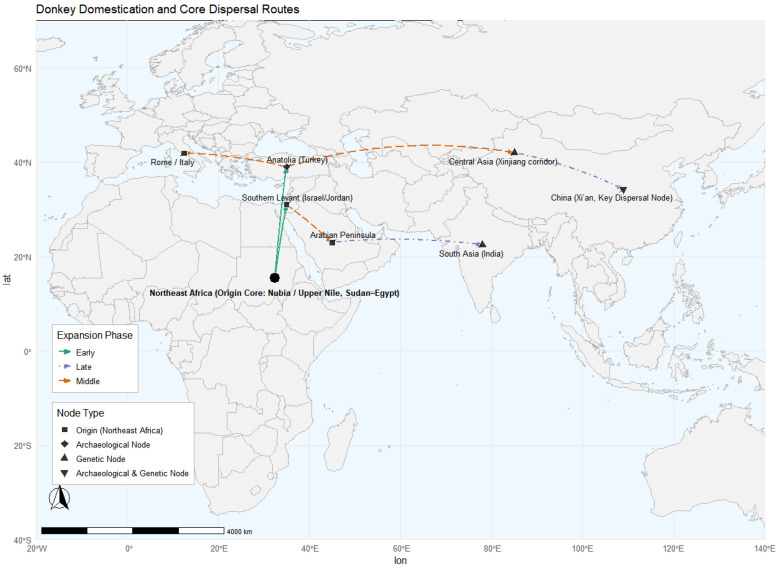
Reconstruction of donkey domestication centers and principal dispersal routes based on integrated archeological and genetic evidence [2,3,26,55,56]. Colored dashed lines indicate different expansion phases. Node shapes denote evidence types: squares, origin center (Northeast Africa); diamonds, archaeological nodes; upward triangles, genetic nodes; and inverted triangles, nodes supported by both archaeological and genetic evidence.

**Figure 3:**
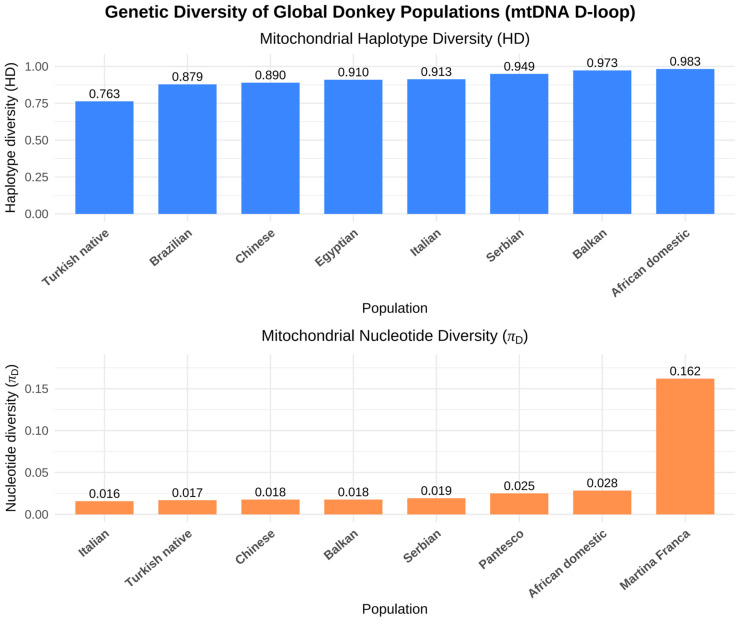
Global distribution of mitochondrial DNA D-loop haplotype diversity in donkey populations. Analysis based on mtDNA D-loop sequences compiled from published datasets [55,57,58,59,60,61,62,63].

**Figure 4:**
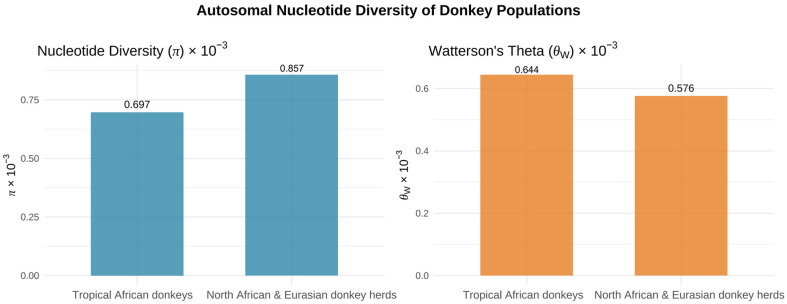
Autosomal nucleotide diversity patterns across African and Eurasian donkey populations. Data derived from [26].

Recent population genomic studies have profoundly expanded our understanding of the evolutionary origins and diversification of domestic donkeys (*Equus asinus*) [12]. Figure 2 illustrates the reconstruction of donkey domestication centers and principal dispersal routes based on integrated archeological and genetic evidence. High-coverage resequencing across global populations has revealed complex demographic histories shaped by multiple dispersal events, recurrent gene flow, and region-specific adaptation [50,51]. The global distribution of mitochondrial DNA D-loop haplotype diversity is shown in Figure 3, based on mtDNA D-loop sequences compiled from published datasets. African donkeys generally retain the highest genomic diversity, reflecting their proximity to ancestral wild populations, whereas many Asian and European populations exhibit reduced heterozygosity—likely a consequence of founder effects, demographic contractions, and prolonged artificial selection [52,53,54]. Figure 4 presents autosomal nucleotide diversity patterns across African and Eurasian donkey populations, highlighting variation derived from genome-wide data. Despite these insights, the global population structure of donkeys remains only partially resolved due to uneven sampling, especially from Africa, and limited integration between genomic, archeological, and historical evidence.

### 4.1. Genomic Signatures of Adaptation and Selection

Genome-wide selection scans in donkeys have identified candidate loci associated with thermoregulation, osmoregulation, metabolism, and skeletal morphology, providing molecular evidence for adaptation to arid and high-temperature environments and for selection on locomotor efficiency and load-bearing capacity during domestication [64,65,66]. Ancient DNA (aDNA) analyses further place the initial domestication of donkeys in northeastern Africa approximately 5000–7000 years before present, broadly consistent with archeological records [1]. However, discrepancies between archeological interpretations and molecular clock estimates persist, likely reflecting challenges related to degraded aDNA and limited sample sizes. Importantly, although many selection signals have been consistently detected across studies, their biological effects remain largely inferred from genomic correlations. Future work should therefore prioritize functional validation using in vitro systems and model organisms to move beyond association-based evidence and to identify causal genes and mechanisms underlying key adaptive and production-related traits.

### 4.2. Multi-Origin Domestication and Genetic Admixture

Comparative analyses of ancient and modern donkey genomes reveal a far more dynamic domestication history than previously assumed [2]. Rather than reflecting a single origin, high-coverage African aDNA demonstrates continuity with extant *Equus africanus* populations, while Eurasian archeological genomes contain signatures of later demographic turnover, recurrent introgression, and population replacement [2]. These patterns indicate that donkeys were repeatedly dispersed and reshaped through human-mediated exchange across transcontinental trade networks such as the Red Sea and Silk Road routes [67,68,69]. Importantly, recent time-calibrated nuclear genome analyses also reveal that domestication involved not only repeated dispersal and admixture events but also a series of region-specific demographic shifts that shaped population structure over time—patterns that were not captured by earlier two-clade domestication models [28].

Uniparental markers contribute complementary perspectives but also highlight the need for genome-wide resolution. Mitochondrial haplogroups (Clades I and II) point to at least two maternal domestication events [56], while the extremely reduced Y-chromosomal diversity reflects strong male-mediated bottlenecks consistent with early breeding systems [54]. However, these markers offer limited power to capture the functional consequences of admixture and selection [70]. Recent integrative frameworks combining uniparental markers, high-resolution nuclear genomes, and calibrated aDNA now reveal directional gene flow among regional lineages and identify functional regions shaped by domestication-related pressures [31,34,71]. This emerging evidence moves the field beyond simple phylogenetic reconstruction toward understanding how demographic processes and selection jointly shaped the genomic architecture of modern donkeys.

## 5. The Genomic Basis of Economic Traits in the Donkey

Recent genomic studies have increasingly elucidated the genetic architecture underlying production, adaptive, reproductive, and dairy-related traits in donkeys. These traits are often interconnected rather than independent, with overlapping genetic control influencing growth, thermotolerance, metabolic efficiency, reproductive performance, and milk traits [2,26,33,50,72].

### 5.1. Production and Adaptive Traits

Donkey populations from Africa, the Mediterranean, and China display pronounced variation in body size, musculature, and skin characteristics, reflecting adaptation to diverse ecological and husbandry conditions. Genomic studies have repeatedly highlighted *LCORL–NCAPG* and *IGF1* as associated with growth and body size, where *LCORL* promotes limb and body elongation through transcriptional regulation of growth-related pathways, *NCAPG* modulates cell proliferation and muscle fiber hypertrophy, and *IGF1* enhances chondrocyte and myoblast differentiation to drive skeletal and muscular development [73,74]. Pigmentation and skin-structure genes such as *MC1R*, *ASIP*, and *TBX3* influence coat color and dermal traits, with *MC1R* and *ASIP* regulating eumelanin–pheomelanin switching and *TBX3* contributing to hair follicle patterning and epidermal development [26,75,76]. Genes regulating lipid metabolism and muscle fiber development also contribute to carcass composition and meat quality, partly through modulation of intramuscular fat deposition, oxidative vs. glycolytic fiber ratios, and postmortem muscle biochemical properties [77,78]. Positive selection signals in genes involved in osmoregulation, thermotolerance, and metabolic efficiency across donkey populations suggest that renal ion transport, heat-shock protein activity, and mitochondrial energy pathways have been recurrent targets of adaptation, indicating that adaptive capacity and production traits may have co-evolved under combined natural and artificial selection pressures [34,79,80]. Table 1 summarizes the key candidate genes and their associated phenotypic traits identified through donkey genomics research. Nevertheless, most associations remain statistical rather than functionally validated, and differences in trait definitions across studies limit direct comparisons.

### 5.2. Reproductive and Dairy Traits

Reproductive traits constitute an essential component of donkey economic value. Candidate genes such as *GDF9* and *BMP15* and their downstream signaling pathways play key roles in oocyte development and folliculogenesis, where *GDF9* promotes granulosa cell proliferation and cumulus expansion through *SMAD2/3*-mediated signaling, and *BMP15* regulates follicle growth, ovulation rate, and oocyte maturation by modulating granulosa cell differentiation and *FSH* sensitivity [91,93]. While candidate gene and transcriptomic analyses provide insights for genetic improvement of reproductive traits, low heritability and strong environmental effects constrain short-term genetic gains [33,94,95]. Effective enhancement of reproductive performance requires combining genomic prediction with optimized management strategies, including estrus synchronization, artificial insemination, and targeted nutritional interventions [96,97].

**Figure 5:**
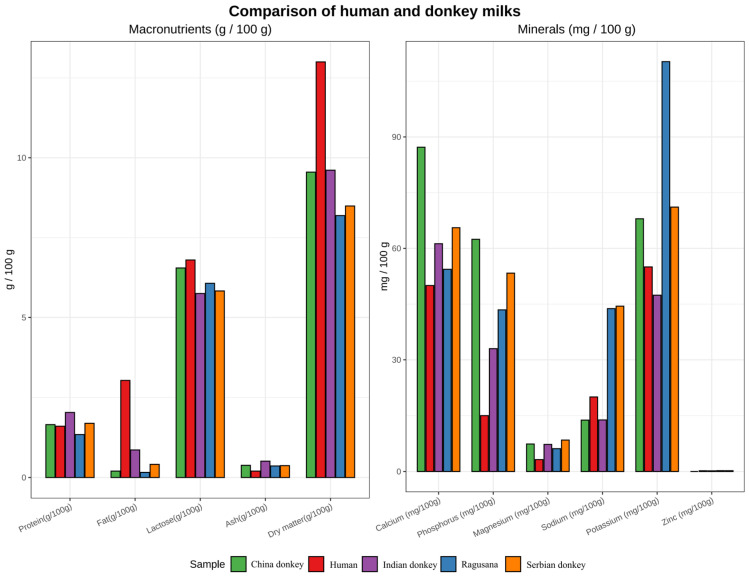
Comparative compositional analysis of macronutrients and minerals in donkey milk from different breeds relative to human milk. Data were compiled from published literature sources [105,106,107,108,109,110,111,112,113], including multiple donkey breeds and human reference datasets. Key macronutrients (protein, fat, and lactose) and major minerals are shown using standardized concentration metrics on a comparable scale. The figure was generated using R (version 4.4.1) based on literature-derived datasets.

Donkey milk exhibits a distinctive composition characterized by a low casein-to-whey protein ratio and elevated concentrations of lactose and lactoferrin, resulting in biochemical and bioactive properties that closely resemble human milk [98,99]. Figure 5 presents a comparative compositional analysis of macronutrients and minerals in donkey milk from different breeds relative to human milk. Genetic determinants influencing milk composition, including variants in the *CSN* gene family and copy number variations in *LYZ*, represent potential targets for selection, with *CSN* gene variants affecting casein micelle formation, protein stability, and digestibility, and *LYZ* copy number influencing lysozyme expression and antimicrobial activity in milk [100,101,102,103]. However, standardized lactation phenotyping and functional validation across breeds remain limited [104].

### 5.3. Multi-Omics Integration and Functional Verification of Candidate Loci

**Figure 6:**
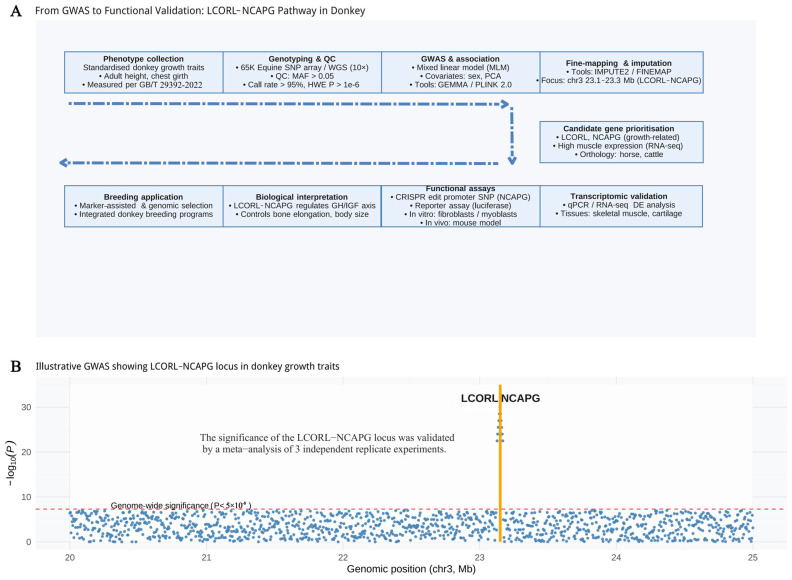
From genome-wide association analysis to functional verification: research framework and GWAS visualization of the LCORL–NCAPG pathway in donkey growth traits. Growth-related phenotypes were measured following **China National Standard GB/T 29392-2022** and common livestock measurement protocols [125]. The content is derived from published studies [2,24,26,31,38,124,126] based on GWAS results from multiple donkey populations. The figure was visualized using **R (version 4.4.1)**. Horizontal dashed lines indicate genome-wide significance thresholds (*p* < 5 × 10^−8^); vertical lines highlight the LCORL–NCAPG region, supported by meta-analysis of three independent replicates.

Multi-omics integration is essential for converting statistical associations into mechanistic insights of candidate genes in donkeys. Rather than relying on single-layer analyses, effective workflows combine genome-wide association studies (GWAS) or genome-wide selection scans with tissue-specific transcriptomics (RNA-seq), epigenomics (ATAC-seq, ChIP-seq), proteomics, and metabolomics [80,114,115,116,117,118,119,120,121,122]. Figure 6 shows a simulated framework from GWAS to functional verification of the *LCORL–NCAPG* pathway. Transcriptomic and epigenomic data reveal tissue- or stage-specific gene regulation, while proteomic and metabolomic layers capture downstream functional consequences of genetic variation [115,117,120]. Integrating these layers enables the identification of causal alleles, functional networks, and biomarkers for traits of interest. Nevertheless, most donkey studies still rely on low-coverage whole-genome resequencing in geographically restricted, small populations, which reduces statistical power, increases false-positive associations, and limits replication and functional validation [27,123,124]. These constraints hinder the translation of genomic discoveries into practical breeding or conservation strategies.

To overcome these limitations, donkey studies should adopt rigorous experimental designs, including increasing sample sizes where feasible, standardizing phenotyping protocols, recording environmental covariates, and applying statistical corrections for population structure and relatedness [127]. Multi-omics data can be integrated into a unified functional genomics framework: GWAS or selection scans define candidate loci (“genomic coordinates”), transcriptomic and epigenomic data reveal tissue- and context-specific expression, and proteomic or metabolomic layers provide evidence of downstream functional effects [115,128]. Causal inference approaches, such as gene regulatory network reconstruction or Mendelian randomization, further strengthen mechanistic interpretation [129,130]. Applying this integrative framework in donkeys enables systematic validation of candidate loci, clarifies allele-to-trait relationships, and supports evidence-based genomic selection strategies that optimize adaptive or production traits while preserving genetic diversity.

## 6. Applications of Genomics in Donkey Breeding and Conservation

Genomic technologies are increasingly applied to donkey breeding and conservation management [26,51]. Beyond describing population structure, recent genomic analyses provide actionable insights that can guide selection, maintain diversity, and enhance adaptive potential. GWAS and whole-genome resequencing across multiple populations have identified genomic regions associated with body size, coat color, metabolic efficiency, and reproductive traits [51,80]. For example, Dezhou and Yangyuan donkeys in China exhibit significant SNP signals linked to growth and pigmentation, whereas the Martina Franca and Ragusano breeds in Italy show localized selection in genes influencing body conformation and reproduction [33,36,50]. These findings offer a genomic foundation to inform strategic breeding and conservation plans.

To translate genomic insights into practical management, population-level indicators such as Runs of Homozygosity (ROH), genomic inbreeding coefficients, and nucleotide diversity can be incorporated into genomic mating programs that minimize relatedness, prevent excessive homozygosity, and preserve rare haplotypes [131,132,133]. Conservation units can be prioritized using diversity-based genomic ranking, emphasizing low-inbreeding maternal lines and implementing controlled outcrossing for high-ROH lineages [16]. Beyond mating decisions, genomic data can guide nutrition to enhance reproduction; for example, in Martina Franca jacks, hemp-based PUFA supplementation improved membrane lipids and fertility [134]. This example highlights the practical translation of genomic knowledge into farm-level interventions that optimize breeding performance. However, the routine application of whole-genome sequencing or high-density SNP arrays remains economically challenging in many low-income regions where donkeys are most critical to livelihoods, as per-sample costs can be an order of magnitude higher than locally sustainable breeding budgets. In this context, low-density SNP panels combined with imputation or ddRAD-seq genotyping offer cost-effective alternatives, enabling population monitoring, nutritional guidance, and inbreeding control at a fraction of the cost while retaining sufficient resolution for management decisions [50]. Integrating these genomic metrics with phenotypic and pedigree data further improves breeding value estimation and supports marker-assisted management of economically important loci [33,50].

Comparative genomic studies reveal pronounced geographic differentiation among donkey populations, reflecting divergent evolutionary histories and management practices. African donkeys, including Abyssinian, Afar, and other breeds from the Horn of Africa and East Africa, harbor high genomic diversity and adaptive alleles associated with thermotolerance, drought resilience, and immune function, consistent with long-term exposure to arid and variable environments [2,135,136]. However, African populations remain underrepresented in current genomic datasets, and future sampling efforts should incorporate additional indigenous lineages to improve demographic resolution and capture the full spectrum of ancestral diversity. Mediterranean donkeys, particularly semi-feral or traditionally managed breeds such as Pantesco and Amiatina, exhibit moderate genetic diversity and clear population structuring shaped by regional selection on metabolism, morphology, and local environmental adaptation [50]. In contrast, intensively selected Chinese breeds, including Dezhou and Liangzhou, show reduced genomic diversity, extended runs of homozygosity, and strong selective sweeps targeting growth- and reproduction-related pathways, reflecting recent directional selection under modern breeding systems [26,33,123]. Recent studies integrating whole-genome resequencing with RNA-seq-based functional annotation further indicate that some breed-specific sweeps are driven by regulatory rather than coding variation, underscoring the value of integrative approaches for distinguishing adaptive signals from demographic effects [79,137]. Together, these patterns highlight that donkey genetic improvement and conservation strategies should be population-specific and context-sensitive rather than globally uniform [138]. For clarity, the principal advantages and practical constraints associated with different genomic approaches are summarized in Table 2.

## 7. Challenges, Limitations, and Ethical Considerations

### 7.1. Phenotypic Data Quality and Standardization

The utility of donkey genomics is constrained by fragmented datasets, variable sequencing quality, and inconsistent bioinformatic pipelines across studies [26,29,133,136]. Such technical heterogeneity limits cross-study comparability and reduces the accuracy and transferability of genomic predictions [146,147]. Donkey-specific features further exacerbate these challenges, as generally small and geographically isolated populations intensify genetic drift and inbreeding, thereby diminishing the statistical power of association analyses and genomic selection models [50,148,149].

In parallel, phenotypic data remain a major bottleneck. Measurements of growth, reproduction, and milk traits are frequently incomplete or non-standardized, with protocols differing widely across regions, management systems, and breeds [24,33,150]. This lack of harmonized phenotyping severely limits reliable genotype–phenotype integration and the downstream application of genomic tools [12,151]. Establishing shared reference populations, standardized phenotyping guidelines, and transparent data curation frameworks will be essential to improve reproducibility and analytical robustness.

### 7.2. Technical, Resource, and Policy Constraints

Progress in donkey genomics is often restricted by limited institutional capacity, insufficient funding, and uneven access to sequencing and computational infrastructure [32,120,152]. These constraints slow the expansion of population-scale genomics and multi-omics integration, particularly in regions where donkeys are most socioeconomically important.

Although international frameworks such as the Nagoya Protocol aim to promote ethical and equitable exchange of genetic resources, their implementation can create practical barriers to collaboration in low-resource settings. Complex permitting procedures, extended approval timelines, and administrative reporting requirements may delay or restrict material transfer and data sharing when local infrastructure and regulatory support are limited [153]. Targeted investment in regional genomic facilities, training programs, and interoperable open-access platforms will be critical to reduce these disparities and enable balanced global participation in donkey genomic research and conservation.

### 7.3. Ethical and Biosafety Considerations

Emerging biotechnologies, including genomic selection and genome editing, offer theoretical potential to improve disease resistance and reproductive performance in mammals [154,155,156]. However, their application in donkeys raises species-specific ethical and biosafety concerns. In endangered or locally adapted populations, genome editing may unintentionally disrupt population structure, accelerate inbreeding, or compromise adaptive traits shaped by long-term environmental and cultural selection [157,158].

Given the multifunctional roles of donkeys in transport, agriculture, and livelihoods, any welfare, behavioral, or metabolic consequences of genetic intervention must be carefully assessed [159]. Evidence from other livestock species—such as metabolic imbalances following MSTN knockout in pigs [160,161]—illustrates risks that would be unacceptable in small or vulnerable donkey populations. In addition, gene flow between edited donkeys and wild or semi-feral groups could threaten the genetic integrity of local ecotypes, particularly in regions where domestic and wild lineages coexist [26].

To mitigate these risks, future research should prioritize donkey-specific genomic resources, controlled in vitro functional validation, and conservation-oriented breeding frameworks [162]. Any application of advanced biotechnologies should proceed under transparent governance, strict welfare oversight, and long-term genetic monitoring to balance innovation with preservation.

## 8. Conclusions and Perspectives

Donkey genomics has made significant strides in recent years, producing chromosome-level reference genomes, population-scale resequencing datasets, and multi-omics analyses that offer valuable insights into the species’ evolutionary history, population structure, and the genetic underpinnings of important traits such as growth, reproduction, milk production, and environmental adaptation. Research has revealed that adaptive traits, such as thermotolerance and drought resilience, often evolve in conjunction with production traits, demonstrating the interconnected genetic basis of both fitness and performance. These findings highlight the complexity of donkey genetics and emphasize the need for a nuanced approach to breeding and conservation efforts.

While genomic tools have the potential to quickly influence donkey population structure, it is crucial to balance the maintenance of genetic diversity with the targeted enhancement of key traits. Research indicates that modulating gene expression at selected loci, while preserving effective population sizes, can improve both adaptive and production traits without causing significant loss of diversity. However, excessive directional selection in small or intensively managed populations risks depleting rare haplotypes and locally adapted alleles. To advance practical applications in breeding and conservation, future research should focus on expanding genomic datasets across diverse populations, implementing high-resolution phenotyping, validating candidate loci, and developing cost-effective genomic resources to support informed breeding programs and conservation strategies. This approach will help bridge theoretical genomics with applied practices to ensure sustainable improvements in donkey populations.

## Figures and Tables

**Table 1:** Summary of key candidate genes and their associated phenotypic traits identified through donkey genomics research.

**Category**	**Gene(s)**	**Breed(s)/Study System**	**Reported Association/Functional Note**	**Reference**
Growth	*NCAPG–LCORL* *TMEM154*	Multiple Chinese donkey populations (including Biyang, Sichuan, Liangzhou, and Hetian Gray)	Identified as strong candidate genomic regions associated with chest circumference and overall body size; the function of *TMEM154* has been experimentally verified.	[48]
Growth	*POLR2A* *CHRNB1* *FGF11* *ZBTB4*	Dezhou donkey	Candidate genes associated with body development-related traits.	[81]
Growth	*NFATC2* *PROP1* *UBB* *HAND2*	Xinjiang donkey	Candidate genes influencing chest circumference and body conformation traits.	[82]
Growth	*COG6*	Hetian Gray donkey	Newly identified candidate gene associated with donkey body shape.	[34]
Milk	*CSN gene family*	Amiata donkey	Genes affecting milk protein composition and processing characteristics.	[83]
Milk	*LYZ*	Ragusano and Grigio Siciliano donkeys	Associated with antimicrobial activity in milk by inhibiting pathogen growth; the function of *LYZ* has been experimentally verified.	[84]
Milk	*CSN3* *LTF*	Turkish donkey	Genes associated with milk production and related traits.	[85]
Milk	*TOP1MT* *GPIHBP1* *DRG2* *FLII* *PLK1*	Xinjiang donkey	Candidate genes related to average daily milk yield and total milk production.	[86]
Coat color	*MC1R*	Seven French studbook donkey breeds and American miniature donkeys	Allelic variation in *MC1R* is associated with red, chestnut, and black coat color phenotypes; gene function has been experimentally verified.	[87]
Coat color	*ASIP*	Dezhou donkey	Gene affecting coat color variation in mammals; the function of *ASIP* has been experimentally verified.	[36]
Coat color	*KIT* *TYRP1*	Feral Asinara white donkeys (Italy) and multi-breed donkey populations showing white or white-spotted phenotypes (e.g., Miniature Donkey, Mammoth Jack).	Genes correlated with coat color and hair type traits.	[88,89]
Reproduction	*WEE2*	Chinese Biyang donkey	Gene associated with oocyte development in donkeys.	[90]
Reproduction	*CNBP* *CCNB1* *CENPE*	Chinese Biyang donkey	Genes involved in the maintenance of oocyte maturation.	[91]
Adaptation	*HSP70*	Donkeys in Nigeria	Gene associated with heat stress response and thermal adaptation.	[92]
Adaptation	*EPAS1* *FAM184B*	Multiple Chinese donkey populations (including Biyang, Sichuan, Liangzhou, and Hetian Gray)	Candidate genes involved in high-altitude adaptation.	[48]
Adaptation	*GLDC* *HBB*	Dezhou donkey, Qinghai donkey, Guola donkey, Guanzhong donkey, Kulun donkey and Xinjiang donkey	Genes associated with adaptation to hypoxic (low-oxygen) environments.	[79]

**Table 2:** Advantages and constraints of applying genomic tools in donkeys.

**Genomic Tool/Application**	**Main Advantages**	**Main Constraints**
Chromosome-level reference genomes	Provide high-quality gene annotation and structural variation information; enable accurate identification of candidate genes and regulatory elements [28,37].	High construction cost; limited representation of local breeds and wild relatives in some references [2].
Population-scale whole-genome resequencing (WGS)	Resolves population structure, selection signatures, and rare variants at high resolution; allows simultaneous assessment of genetic diversity and selection intensity [49,139].	High sequencing and computational costs; limited feasibility in low-income regions [123].
Genome-wide association studies (GWAS)	Identify genomic regions associated with growth, reproduction, and adaptive traits; support marker-assisted management [33,82].	Highly sensitive to phenotyping quality and sample size; limited power for complex traits [33,86].
Runs of Homozygosity (ROH) and genomic inbreeding analyses	Quantify inbreeding levels and historical bottlenecks; identify high-risk lineages [65,140]	Require medium- to high-density genotype data; threshold definitions may vary among populations [141].
Low-density SNP panels with imputation	Cost-effective and operationally feasible; suitable for large-scale monitoring and long-term management [123].	Lower resolution than WGS; imputation accuracy depends on reference panels [123,142].
Reduced-representation sequencing (e.g., ddRAD-seq)	Economically efficient; suitable for multi-population comparisons and diversity assessments [50].	Uneven genomic coverage; limited cross-study comparability [50].
Multi-omics integration (e.g., RNA-seq)	Reveals regulatory mechanisms underlying phenotypic and adaptive variation; distinguishes regulatory from coding selection [90,143,144].	Experimentally and analytically demanding; high requirements for sample quality [6,45].
Genomics-assisted breeding and management	Improves selection efficiency; enables informed decisions at individual and population levels [145].	High demands on technical infrastructure, expertise, and sustained funding [145].

## Data Availability

All the data are available in the manuscript.
